# Socheongryong-tang for improving nasal symptoms associated with allergic rhinitis: A study protocol for a randomized, open-label, cetirizine controlled, clinical trial

**DOI:** 10.1097/MD.0000000000011812

**Published:** 2018-08-24

**Authors:** Young-Eun Kim, Mi Ju Son, So Young Jung, Ojin Kwon, Jun-Hwan Lee, Dong-Hyo Lee

**Affiliations:** a Korea Institute of Oriental Medicine, Yuseong-gu, Daejeon; b Department of Korean Preventive Medicine, College of Korean Medicine, Graduate School, Kyung Hee University, Dongdaemun-gu, Seoul; c Korean Medicine Life Science, University of Science and Technology, Yuseong-gu, Daejeon; d Department of Ophthalmology and Otolaryngology and Dermatology, Woo-Suk University Korean Medicine Hospital, Wansan-gu, Jeonju-si, Jeollabuk-do, Republic of Korea.

**Keywords:** allergic rhinitis, cetirizine, herbal medicine, randomized controlled trial, Sho-seiryu-to, Socheongryong-tang, Xiao-Qing-Long-Tang

## Abstract

**Introduction:**

Socheongryong-tang (SCRT) is an herbal medicine with anti-inflammatory and anti-allergic properties, commonly used in East Asian countries to reduce rhinitis symptoms. There have been several clinical studies of its effects on allergic rhinitis (AR), but no trials comparing it with conventional treatment. We present the protocol for a feasibility trial to assess the safety and clinical effectiveness of SCRT in AR in comparison with cetirizine.

**Methods and analysis:**

This is a randomized, open-label, cetirizine-controlled clinical trial. A total of 30 AR patients who have signed informed consent forms will be recruited and randomly assigned to SCRT or cetirizine groups at a 1:1 ratio. The participants will visit the clinical research center every week and receive SCRT granules or cetirizine tablets. SCRT will be taken twice daily, cetirizine will be taken once daily, and treatment medication will be taken for 2 weeks. Data will be collected at baseline, at week 2, and at week 4 after random allocation. The primary outcome will be the mean change in the total nasal symptom score from baseline to week 2. Secondary outcome measures will include the mini Rhinoconjunctivitis Quality of Life Questionnaire and total serum immunoglobulin E. To assess the safety of SCRT, a liver and renal function test will be conducted before and after treatment, and the participants will be asked about any occurrence of adverse events at every visit. The recruitment rate, completion rate, and medication adherence will also be calculated to assess feasibility.

**Discussion:**

The findings of this study are expected to provide the basis for a full-scale randomized controlled trial to confirm the safety and effectiveness of SCRT for the treatment of nasal symptoms in patients with AR patients not controlled by conventional therapy.

**Trial registration:**

This study has been registered at the Korean National Clinical Trial Registry, Clinical Research Information Service (KCT0002380).

## 1 Introduction

Allergic rhinitis (AR) is an inflammatory disease caused by an immunoglobulin E (IgE)-mediated response and is characterized by one or more typical nasal symptoms of rhinorrhea, nasal congestion, sneezing, and nasal itching, as well as non-nasal symptoms such as tears, ocular hyperemia, itching around the eyes, and itchy palate.^[1,2]^

AR is a common condition, and the prevalence has been increasing over the last decade. The prevalence of AR is 10% to 30% of the world's population; the prevalence is 11.9% to 30.2% in the United States^[3]^ and around 22.7% in European countries.^[4]^ Although the nasal symptoms of AR are not threatening to life, the symptoms can affect social and daily activities such as sleep, learning, and work, ultimately generating considerable medical expenses.^[5,6]^ Moreover, when AR occurs repeatedly, it becomes difficult to treat and can cause complications such as bronchial asthma, rhinosinusitis, conjunctivitis, and otitis media.^[7]^

The general treatment and management of AR aims at relieving symptoms quickly by avoiding the causes of allergens and managing symptoms to minimize recurrence.^[8]^ However, most people cannot completely avoid allergens due to industrialization, modernization, and urbanization of residence.^[9–12]^ Conventional therapies such as antihistamines, decongestants, and glucocorticoids are used to control AR symptoms, but there are the risks of adverse effects with long-term use, and the effects do not persist when the medication is stopped. Moreover, some AR patients cannot take medication because of its side effects.^[13]^

Due to the limited success of conventional medical treatments, various complementary and alternative medicines are currently used in clinical settings. In East Asian countries, complementary and alternative medicine in the form of traditional herbal medicine is widely used to treat AR.^[14]^ Socheongryong-tang (SCRT, known as Sho-seiryu-to in Japanese, Xiao-Qing-Long-Tang in Chinese) is an herbal formula broadly used throughout East-Asian countries,^[15]^ and is approved by the Korean Ministry of Food and Drug Safety (MFDS) for bronchitis, asthma, rhinitis, and cold-related symptoms including rhinorrhea and cough.^[16]^ According to previous studies, SCRT has anti-inflammatory and anti-allergic properties.^[17,18]^ A survey of Korean physicians found that SCRT is the most widely used herbal prescription for the treatment of AR.^[19]^ There is evidence that SCRT is more effective than placebo in reducing nasal symptoms associated with AR.^[20]^ However, there is no evidence that SCRT is a viable alternative to conventional therapies, especially for AR patients whose symptoms are not controlled by conventional medication.

Antihistamines are the first-line treatment in AR.^[21]^ Cetirizine (Zyrtec Tab, Korea UCB Co., Ltd. Seoul, Korea) was selected as the control for this study because it is the most commonly used antihistamine.^[22]^ Cetirizine is a specific and long-acting histamine H1 receptor antagonist. It has marked anti-allergic properties and inhibits eosinophil chemotaxis during the allergic response, and has shown efficacy and safety in the treatment of patients with AR.^[23]^

The purpose of this study is to validate a protocol for comparing the effectiveness of SCRT with that of conventional medicine. The findings of this study are expected to provide a base for large-scale randomized controlled trials of the comparative effectiveness of SCRT for the treatment of AR, and may consequently serve to improve future treatment strategies for this condition.

## 2 Methods

### 2.1 Objectives

This study is a feasibility trial to assess the safety and clinical effectiveness of SCRT on the AR, in comparison with cetirizine.

### 2.2 Trial design and setting

This is a randomized two-parallel-arm open-label cetirizine-controlled comparative effectiveness study. This study will be conducted at Woosuk Korean Medicine Medical Center, Jeonju, Republic of Korea. The study has 2 arms: the SCRT treatment group and the cetirizine control group. A total of 30 participants will be recruited for this trial. Cetirizine will be used as an active control to compare the effectiveness of SCRT on AR patients whose symptoms are not controlled by conventional medication. The total study period will be 5 weeks. It consists of a week run-in period after screening, 2 weeks of medication, and 2 weeks of follow-up after medication is stopped.

**Figure 1:**
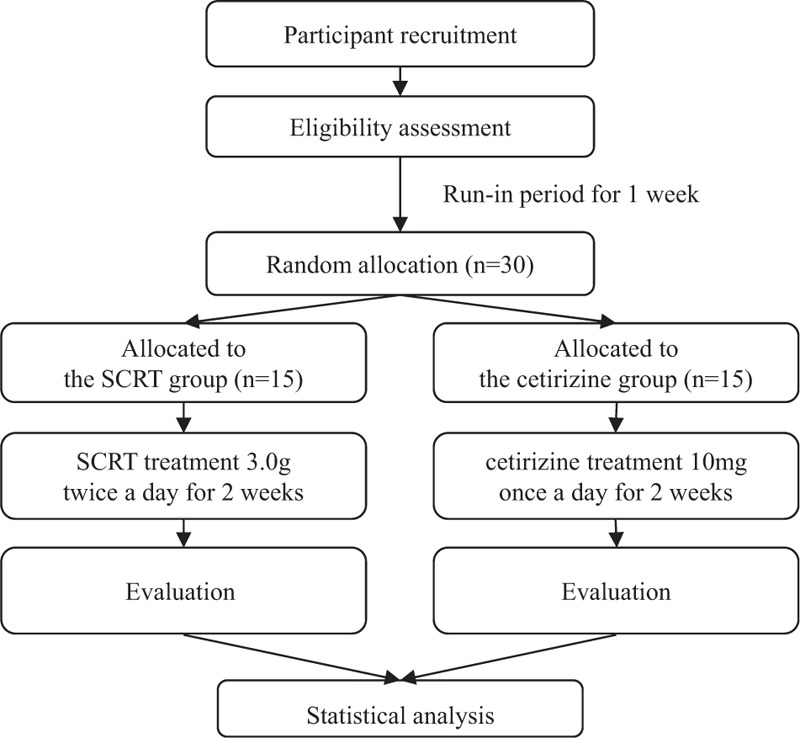
Flowchart describing the study plan. SCRT = Socheongryong-tang.

The detailed design is summarized in Fig. 1 and Table 1. The study protocol (version 1.3) follows the Standard Protocol Items: Recommendations for Interventional Trials (SPIRIT) guidelines (see Additional File 1).

**Table 1:**
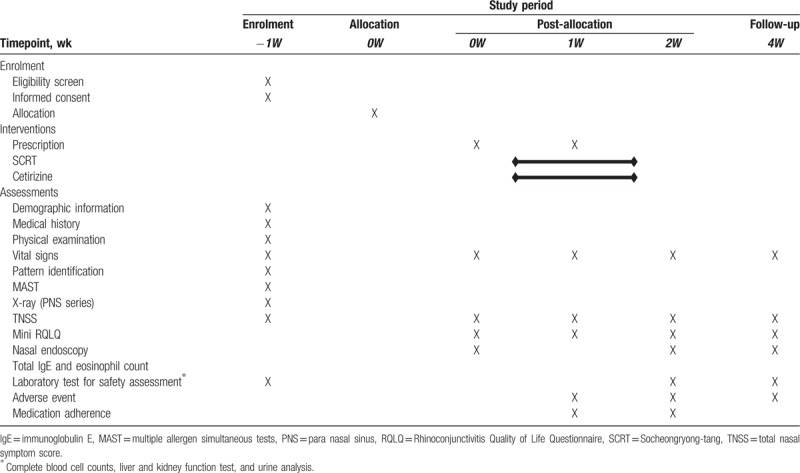
Schedule of enrolment, interventions, and outcome measurements for a randomized controlled trial assessing the safety and clinical effectiveness of SCRT in allergic rhinitis compared with cetirizine.

### 2.3 Recruitment

The trial will be advertised on the hospital bulletin board, local newspapers, and websites used by local community residents. If necessary, potential participants who express a desire to enroll will be provided information about the study or pre-screened for eligibility before the first visiting to the hospital, by clinical trial coordinators via a telephone. Written informed consent will be obtained from all study participants prior to enrolment, and participants may decline to participate or withdraw at any time without disadvantage.

### 2.4 Study participants

The inclusion and exclusion criteria of participation in this study are set according to the Allergic Rhinitis and its Impact on Asthma (ARIA) guideline criteria for persistent AR.

#### 2.4.1 Inclusion criteria

Participants will be included in this study if they meet the following criteria:

(1) Either male or female, 19–65 years of age
(2) Persistent AR patient, meaning all of the following:
  A. Presence of 2 or more symptoms of AR (rhinorrhea, nasal congestion, sneezing, and nasal itching)
  B. Symptoms existing 4 days a week for more than 4 weeks
  C. Persistent AR with a history of more than 2 years, with symptoms not controlled by antihistamine treatment
(3) Positive allergic reaction in the multiple allergen simultaneous test (MAST)
(4) Voluntary participation in this study and signing of the patient consent form after explanation of the purpose of the study.

#### 2.4.2 Exclusion criteria

Participants will not be eligible if they meet one or more of the following criteria:

1. A previous history of nose-related or allergy-related disease and treatment, including any of the following:
  A. Congenital deviated nasal septum, nasal polyps, acute or chronic sinusitis, asthma symptoms
  B. Use of topical decongestants or cromolyn sodium within the previous 3 days
  C. Use of antihistamines or anticholinergics within the previous week
  D. Use of intranasal or oral steroids within the previous 2 weeks
  E. Use of leukotriene receptor antagonists within the previous 4 weeks
  F. Use of Korean medicine treatment (acupuncture, moxibustion, herbal medicine, cupping, etc) for the treatment of AR within the previous month
  G. Receipt of immunotherapy within the previous 5 years
2. Presence of serious medical conditions that could interfere with clinical trial participation, including any of the following:
  A. Hypertension (systolic blood pressure greater than 160 mm Hg or diastolic blood pressure greater than 100 mm Hg)
  B. Diabetes mellitus (fasting blood glucose greater than 126 mg/dL)
  C. Renal dysfunction (serum creatinine level more than twice the normal range)
  D. Liver function abnormality (alanine aminotransferase or aspartate aminotransferase levels that are more than twice the upper limit of the normal range)
  E. Serious hyperlipidemia, anemia, active tuberculosis, thyroid disease, or other serious inflammatory or systemic disease
  F. Past or present history of malignant tumors
3. Pregnancy or breast-feeding
4. Other; ineligible for participation as judged by the investigator:
  A. Those who cannot use mobile applications to record symptoms of AR daily.

After voluntary consent to the study, potential participants will be screened using MAST at the first visit to determine if the rhinitis is allergic. In the MAST, the amount of IgE present in the serum is measured to determine the cause of 92 kinds of respiratory and food allergies and to determine the severity of the allergic reaction.^[24]^ To rule out the possibility of suppression of allergic reaction, drugs containing antihistamines and steroids will be stopped a week before the test. At any time during the clinical trial, the participant may voluntarily withdraw from the clinical trial or can be dropped at the researcher's discretion.

### 2.5 Intervention

Participants will receive SCRT or cetirizine for 2 weeks. Participants in the intervention group will receive SCRT. They will take a packet of the medicine (3.0 g) with water twice a day before breakfast and dinner. Participants in the control group will receive cetirizine. They will take 1 tablet (10.0 mg) before bedtime. The dosage regimen is determined according to MFDS approvals.^[16]^

SCRT (Kracie sho-seiryu-to, K-02) is light brown fine granule with some sour and sweet taste. SCRT will be supplied by Kracie (Kracie Pharmaceutical, Ltd. Tokyo, Japan). A 6.0 g sample of SCRT contains 5.2 g of a dried extract which consists of eight medicinal herbs, *Pinellia tuber* 6.0 g, *Schisandra fruit* 3.0 g, processed ginger 3.0 g, cinnamon bark 3.0 g, *Glycyrrhiza* 3.0 g, *Ephedra herb* 3.0 g, *Asiasarum* root 3.0 g, and peony root 3.0 g. Quality control and quality assurance regarding the identification of products as well as quality and safety testing will be conducted by the manufacturer, which is certified for good manufacturing practices of herbal medicine granules by the Korean MFDS. In qualitative testing, isopropanol, ephedrine, glycyrrhizin, and paeoniflorin are determined in the laboratory of Kracie (Kracie Pharmaceutical, Ltd. Tokyo, Japan) using thin layer chromatography analysis.

Cetirizine is an over-the-counter drug in South Korea. We will record the drugs taken by each participant at every visit to confirm adherence to the protocol. Also, participants will be requested to notify us of any change to their medication/supplement regimen. Additional acupuncture treatments, herbal prescriptions, or therapeutic interventions by other clinicians will not be allowed during the treatment period.

### 2.6 Outcome measures

#### 2.6.1 Primary outcome

The primary outcome is the change in total nasal symptom score (TNSS). TNSS will be recorded in participants’ diaries every day before bedtime using a mobile application. A 4-point severity scale (no symptoms, 0; mild, 1; moderate, 2; and severe, 3) will be used.^[25]^ Using 4 weeks of TNSS scores, we will analyze the differences in the effects of SCRT and cetirizine, and the duration of the effects of each medication.

#### 2.6.2 Secondary outcomes

Secondary outcomes include the mini Rhinoconjunctivitis Quality of Life Questionnaire (RQLQ) and total serum IgE, which will be measured 3 times in total, at screening and 2 and 4 weeks after randomization. The mini-RQLQ is an abbreviated form of the RQLQ, and consists of 14 questions about activities, behavioral problems, nose symptoms, eye symptoms, and other symptoms related to AR. Each item produces a score from 0 to 6 according to the severity of the symptoms.^[26,27]^

To evaluate the effects of SCRT on allergic and inflammatory reactions, we will examine differential white blood cell counts (neutrophil, eosinophil, basophil, monocyte, and lymphocyte) and conduct total serum IgE tests at 2 and 4 weeks after randomization.

To search for the more precise indication of using SCRT, we will use the Cold–Heat Pattern Identification Questionnaire and Nasal Endoscopy Index. The Cold–Heat Pattern Identification Questionnaire is used to distinguish between cold and heat, which is a characteristic of patients from a Korean traditional medicine perspective. The questionnaire consists of 15 symptom-based items, including 8 items related to cold and 7 items related to heat. According to the results of this questionnaire, a patient could be classified as Cold, Heat, neither Cold nor Heat and Cold–Heat complex.^[28]^ The Nasal Endoscopy Index will be used for supplementary evaluation of subjective symptoms of TNSS at the time of randomization and 1, 2, and 4 weeks after randomization. The instrument used is the KAZAMA ENT treatment unit (KAU-3000, HARMONY, ENT Co., Ltd., Incheon, Korea). The efficacy is assessed by scoring the status of the nasal cavity according to an assessment scale developed by Yun et al. It consists of 4 categories for the evaluation of color, dryness/dampness, nasal discharge, and atrophy/edema of the lower nasal turbinates, using a 3-point severity scale scored from normal (0), mild (1), to severe (2).^[29]^

### 2.7 Feasibility assessment

The feasibility of this protocol will be assessed based on the recruitment rate, completion rate, and compliance with medication. The recruitment rate will be presented as a percentage giving the total number of registered participants in proportion to the total number of screened subjects. The completion rate will be presented as a percentage giving the number of subjects who completed the study in proportion to the total number of registered participants. The compliance with medication will be presented as a percentage giving the doses taken by the subjects in proportion to the target doses.

### 2.8 Randomization

Block randomization is performed to assign the same number of subjects to the SCRT or cetirizine group. Thirty participants will be assigned to each group with the same probability that each individual will be selected. An expert on statistics who is not in contact with the participants will generate randomization serial numbers using the statistical program Strategic Applications Software (SAS, V.9.4; SAS Institute, Cary, NC). Before the random assignment, all participants will be informed that they will be allocated to one of 2 groups.

### 2.9 Blinding

To avoid selection bias, the randomization of serial numbers will be managed using sealed envelopes and a locked cabinet. Investigators will open a sealed envelope when a participant is enrolled. The experimental drug in this trial is an herbal medicine that is provided in granules, while the control medication is a drug that comes in tablets. Because patient blinding is not possible, the trial is designed as open label. Although the subjective symptoms of subjects is the main outcome, total serum IgE and Nasal Endoscopy Index will also be examined. The Nasal Endoscopy Index test will be conducted by an investigator who has not been informed about the random assignment and medication and does not know to which group each subject is assigned. This process is intended to reduce the possibility of bias by the evaluators.

### 2.10 Sample size

No clinical trial has been conducted to evaluate the effects of SCRT on the TNSS for patients with AR. Therefore, we adapted the minimum number of subjects recommended for a preliminary study in the field of life sciences, 12 subjects in each group, for 24 subjects in total.^[30]^ Anticipating a dropout rate of 20%, the target of the recruitment is set to 15 subjects in each group, or 30 subjects in total.

### 2.11 Statistical analysis

An independent statistician who is blinded to the randomized allocation of participants will carry out the statistical analysis. Measured variables will be assessed using the full analysis set based on intention-to-treat principles, including all randomized participants who completing the trial without violating any of the exclusion criteria and for whom a complete set of data is available. The per-protocol analysis set will be used for the sensitivity analysis.

The demographic characteristics and baseline data of both groups will be summarized by means and standard deviations for the continuous variables, and frequencies and percentages will be presented for categorical variables. Baseline differences between groups will be assessed using independent *t* tests or Mann–Whitney *U* tests for continuous variables, and the chi-squared test or Fisher exact test for categorical variables. Differences in the primary and secondary outcomes will be analyzed using one-sided independent *t* tests. To demonstrate noninferiority, we will check whether the lower limit of the 2-sided 95% CI of the difference of the primary outcome between the 2 groups is greater than the noninferiority margin. The analysis will be presented as a difference with an associated 95% CI. To determine whether statistically significant differences exist between the groups, differences in the primary and secondary outcomes will be analyzed using a mixed effects model for repeated measurement. Subgroup analyses will also be conducted to investigate the difference in treatment effect according to the type of cold–heat pattern identification questionnaire, each symptoms of AR, and the results of Nasal Endoscopy Index. The level of significance will be set at 0.05 (2-tailed), and all analyses will be performed using SAS (V.9.4; SAS Institute).

### 2.12 Data management

Monitoring will be conducted to control the quality of data and to protect the rights of participants. According to the planned protocol and standard operating procedures, monitors will evaluate whether the recruitment procedures are correctly performed and whether the data are adequately recorded. All data and records related to the clinical trials will be kept in a locked cabinet. Documents related to clinical trials should be kept for 10 years after completion of the clinical trial under the supervision of the custodian after completion of the clinical trial result report.

### 2.13 Safety and adverse events

For observation of the safety of study participants, serum complete blood cell count tests, liver and kidney function tests, and urine analysis will be performed at the screening visit, at the completion of medication and at follow-up 2 weeks after completion of medication. Contact information will be provided to all participants for reporting of adverse events at any time. Investigators will also check vital signs and any expected or unexpected adverse events at every visit. Any adverse events will be reported in accordance with the guidelines of the Institutional Review Board.

### 2.14 Ethics

This study complies with the principles of the Declaration of Helsinki and Good Clinical Practice guidelines. This research protocol has been reviewed and approved by the Institutional Review Board of the Woosuk Korean Medicine Hospital of Woosuk University (WSOH IRB D1705-020-1). This study is registered with the national clinical trial registry Clinical Research Information Service, which is a primary registry of the World Health Organization International Clinical Trials Registry Platform (http://apps.who.int/trialsearch/Trial2.aspx?TrialID=KCT0002380). Voluntary written informed consent will be obtained from all study participants prior to enrolment in the study. If a participant is injured or if adverse drug reactions occur while participating in the study, the subject will be financially compensated under the terms of the insurance contract and the clinical trial center for this study. The knowledge generated from this study will be shared with health care professionals, the general public, traditional medicine associations, and other relevant organizations through manuscript publication, conference presentations, and seminars.

## 3 Discussion

This study is a feasibility trial to assess the safety and clinical effectiveness of SCRT in AR patients who are not well-controlled by conventional therapies.

In this study, we will enroll patients with a history of at least 2 years of persistent AR. The 2 year criterion was chosen based on the inclusion criteria of previous studies of uncontrolled AR patients.^[31,32]^ The strict meaning of “uncontrolled” can be “persistence of severe symptoms,” but in this study we interpreted it as, “the symptom frequently occurs despite steady treatment.” As in previous studies, we considered that 2 years of AR history is long enough to assess the success of conventional therapies. Thus, patients with a history of at least 2 years of persistent AR form the target population in this study.

This study was designed to refine treatment regimen of SCRT, and to evaluate whether SCRT could be an alternative to conventional therapy. In East Asia, SCRT has been used for the treatment of respiratory diseases such as the common cold, asthma, and allergic rhinitis, and studies are being conducted to evaluate its effectiveness.^[20,33,34]^ In this study, we apply cold–heat pattern identification diagnosis, which is a basic diagnostic tool for Korean Medicine.^[35]^ SCRT showed more effectiveness in relieving common cold symptoms in patients who had a cold pattern than in patients who had a hot pattern.^[33]^ Thus, we assume that the effect of SCRT will be different according to cold and heat pattern. By subgroup analysis of cold–heat pattern, we can draw conclusions about the use of pattern identification for prescription of herbal medicine. In addition, we will report the changes of each symptom of allergic rhinitis to find possible indications for SCRT as a complement to cetirizine.

In this study, blinding of the participants is impossible because of the use of different types of drugs (granules for SCRT and tablets for cetirizine). In pragmatic RCTs, although the participants and investigators are often un-blinded, it is still desirable and often possible to blind the assessors or to obtain an objective source of data for evaluation of outcomes.^[36]^ However, in the case of AR, the subjective symptoms of the patient are recommended as the primary outcome. In this study, serum IgE and the Nasal Endoscopy Index, evaluated by 2 independent assessors, are added as a secondary variable to minimize bias.

The findings of this study are intended to provide the basis for a full-scale randomized controlled trial to confirm the safety and effectiveness of SCRT for the treatment of nasal symptoms in AR patients who was not controlled by conventional therapy.

## Author contributions

**Conceptualization:** Dong-Hyo Lee.

**Data curation:** So Young Jung.

**Funding acquisition:** Jun-Hwan Lee, Dong-Hyo Lee.

**Investigation:** Young Eun Kim.

**Methodology:** Mi Ju Son, So Young Jung, Ojin Kwon.

**Writing – original draft:** Young Eun Kim.

**Writing – review & editing:** Mi Ju Son, Dong-Hyo Lee.
